# Benefits of P(LA/CL)‐Hyaluronic Acid Threads With Innovative Hyaluronic Acid Coating Technology (NAMICA)—Preclinical Test Results

**DOI:** 10.1111/jocd.70502

**Published:** 2025-10-16

**Authors:** George Sulamanidze, Dmitri Nikishin, Albina Kajaia

**Affiliations:** ^1^ Limited Liability Company APTOS Tbilisi Georgia; ^2^ Clinic of Plastic and Aesthetic Surgery and Cosmetology “Total Charm Vake” Tbilisi Georgia

**Keywords:** biorevitalization, biostimulation, collagen synthesis, NAMICA technology, P(LA/CL)‐HA threads, skin rejuvenation

## Abstract

**Objective:**

To evaluate the biocompatibility and biostimulatory effects of P(LA/CL)‐Hyaluronic Acid Threads with Innovative Hyaluronic Acid Coating Technology (NAMICA) threads in a porcine model, focusing on collagen synthesis, hydration, and inflammatory response.

**Methods:**

This study utilized a controlled laboratory setting to compare the safety and biostimulation capacity of the new P(LA/CL)‐HA threads produced with NAMICA technology against the previous generation thread. Threads were implanted into hybrid female pigs (Mangalitsa × Duroc), followed by histological analysis at 7, 21, 30, 90, and 180 days post‐implantation to assess collagen types I and III, elastin fibers, and tissue reaction.

**Results:**

P(LA/CL)+HA threads significantly enhanced collagen type I (mean 71.12%, up to 85.90%) and type III (mean 12.18%, up to 20.70%) with minimal inflammation. Elastin fiber content increased over time, with a peak of 2.82% at 180 days in subcutaneous tissue. Capsule thickness around P(LA/CL) threads decreased over time (from 64.28 to 27.23 μm), indicating improved integration.

**Conclusion:**

The P(LA/CL)‐HA threads with NAMICA technology exhibit significant advantages in non‐surgical skin rejuvenation by combining mechanical support with biochemical benefits. They not only lift and volumize but also release hyaluronic acid gradually, enhancing tissue hydration and collagen production while minimizing inflammation. Further studies will validate these benefits in human subjects, potentially establishing new standards for aesthetic treatments.

## 1 Introduction

The natural process of skin aging is an inevitable phenomenon. Various skin rejuvenation products have emerged in modern aesthetic medicine, each offering unique approaches to address the effects of aging.

The effects leading to desirable results for rejuvenation are mechanical (lifting, volumization) and biological (stimulation of extracellular matrix components) [1, 2].

Mesotherapy/biorevitalization with hyaluronic acid (HA) represents one of the most demanded methods with fast‐advancing technology. Hyaluronic acid (HA) is crucial for tissue regeneration and plays a significant role in the modulation of inflammation. Its molecular weight dictates its properties, with high molecular weight HA (HMWHA) having anti‐inflammatory effects and low molecular weight HA (LMWHA) being pro‐inflammatory [3, 4]. Additionally, HA exhibits anti‐inflammatory and antioxidant properties, reducing edema and antagonizing histamine's effects. These findings underscore HA's role in tissue regeneration and inflammation modulation, offering therapeutic potential in aesthetics and beyond [5, 6].

The HA products with diverse polycomponent formulations (vitamins, minerals, amino acids, nucleotides, coenzymes, and antioxidants) currently viable on the market consist of two forms of HA: non‐crosslinked HA (non‐stabilized, an endogenous glycosaminoglycan prevalent in the extracellular matrix) [7] and crosslinked HA (stabilized, where BDDE and DBS are used as stabilizers) [8, 9].

Non‐cross‐linked HA products have been found effective in rejuvenating the skin by improving hydration, increasing collagen synthesis, and enhancing the overall quality of the skin [10]. The short effect of non‐cross‐linked HA‐containing products due to their fast enzymatic degradation is one of the determining reasons for the creation of cross‐linked HA products [9, 11].

Cross‐linked HA is used in fillers [10, 12, 13, 14]. Such fillers primarily contribute to the restoration of volume in areas affected by sagging skin, simultaneously achieving facial rejuvenation and contouring [15].

Face‐lifting threads, made from biocompatible materials like polydioxanone (PDO), polycaprolactone (PCL), or polylactic acid (PLLA), are inserted into the skin to stimulate collagen production while providing immediate lifting. Lifting threads work by enhancing tissue volume, improving sagging areas, and encouraging a more youthful appearance; they induce fibroblast activity, which leads to increased collagen and elastin production, contributing to skin rejuvenation [16, 17]. Unlike traditional fillers, which primarily target specific facial areas, lifting threads offer a broader and more natural result by promoting overall tissue support and regeneration. Different thread types, designed for specific purposes based on their structure, length, and properties, contribute to these effects [18, 19, 20, 21] The ever‐growing experience has led to a surge in innovation from leading thread lift manufacturers. One such innovation is the development of threads incorporating P(LA/CL) and non‐cross‐linked HA. This novel thread composition aims to meet the evolving needs of patients by offering a combination of lifting and biostimulation through enhanced hydration, collagen stimulation, and overall skin rejuvenation.

## 2 Materials and Methods

### 2.1 NAMICA Technology

The breakthrough technology, which by virtue significantly boosts the clinical outcome and reduces adverse side effects during the recovery time, is the creation of a composite from P(LA/CL) and HA in which individual HA particles are fractioned by size, the precisely calibrated percentage ratio, and quantity. Technology itself is the process of transforming the composite from copolymer of poly‐l‐lactide with Σ‐caprolactone and HA into P(LA/CL) nanosized fibers and nano to micro‐sized capsules consisting of hyaluronic acid coated by P(LA/CL) copolymer shell. This unique substance encapsulation technology is called NAMICA (NA—nano, MI—micro, CA—capsules). The first product produced using this technology was the Excellence Visage (EV) threads—core P(LA/CL) monofilament thread covered with micro‐ to nano‐sized capsules with HA as densely as possible (EVHA), allowing the gradual, controlled release of hyaluronic acid for over 6 months post‐thread implantation. The present report deals with the pre‐clinical testing of the EVHA threads produced with NAMICA technology.

**FIGURE 1:**
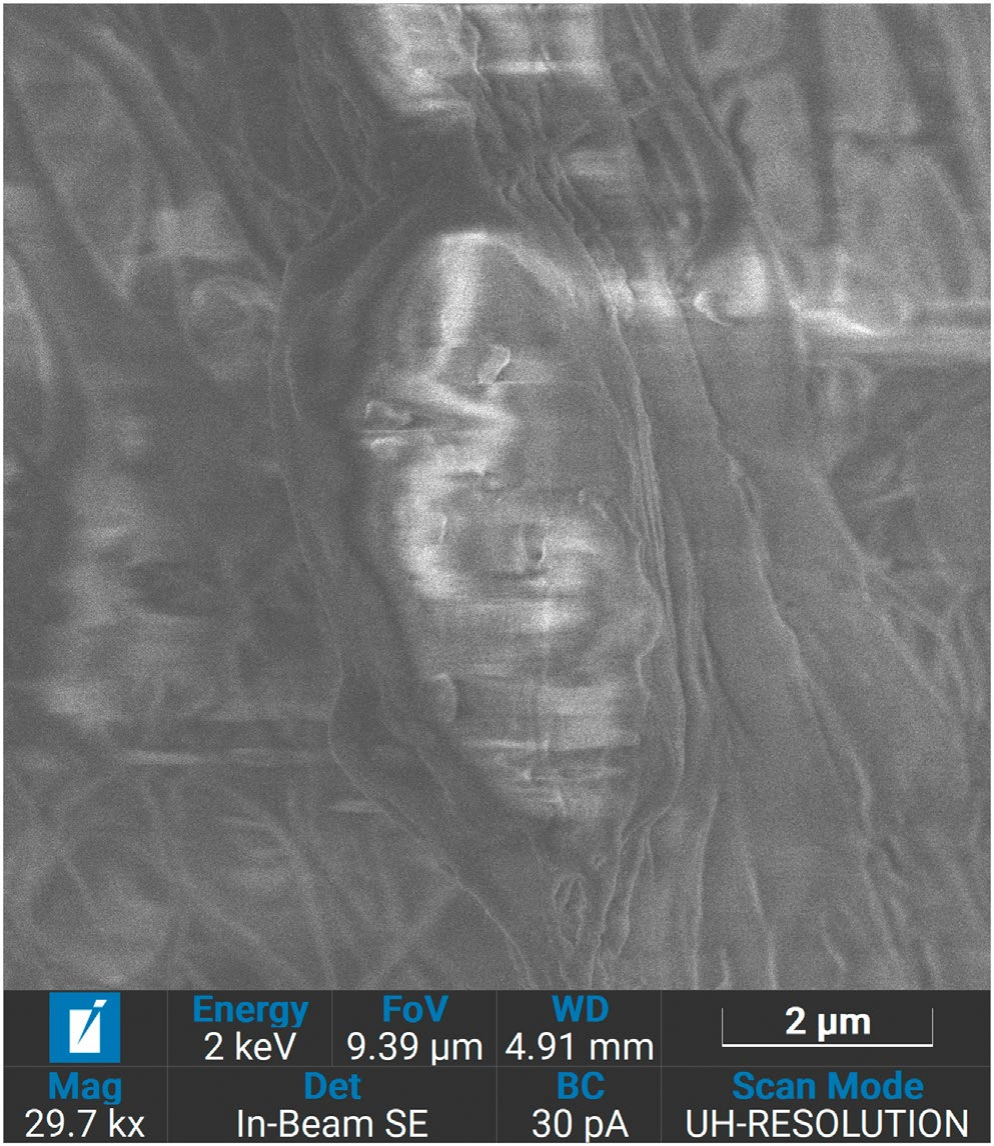
Multiple pores in the shell of the microcapsule. Scanning Electron Microscopy. Magnification ×5000.

It is worth noting that the largest hyaluronic acid spheres or capsules, known as microcapsules, have the thinnest shells. This shell contains pores or fenestrations, which are formed during application due to multidirectional tension forces and the wettability of the particle surface (Figure 1), resulting from the polymerization of the poly‐l‐lactide with Σ‐caprolactone. Through these pores, hyaluronic acid is released into the tissues during the first few hours after thread implantation. The shell of the microcapsules is biodegraded within the first month due to its minimal thickness.

**FIGURE 2:**
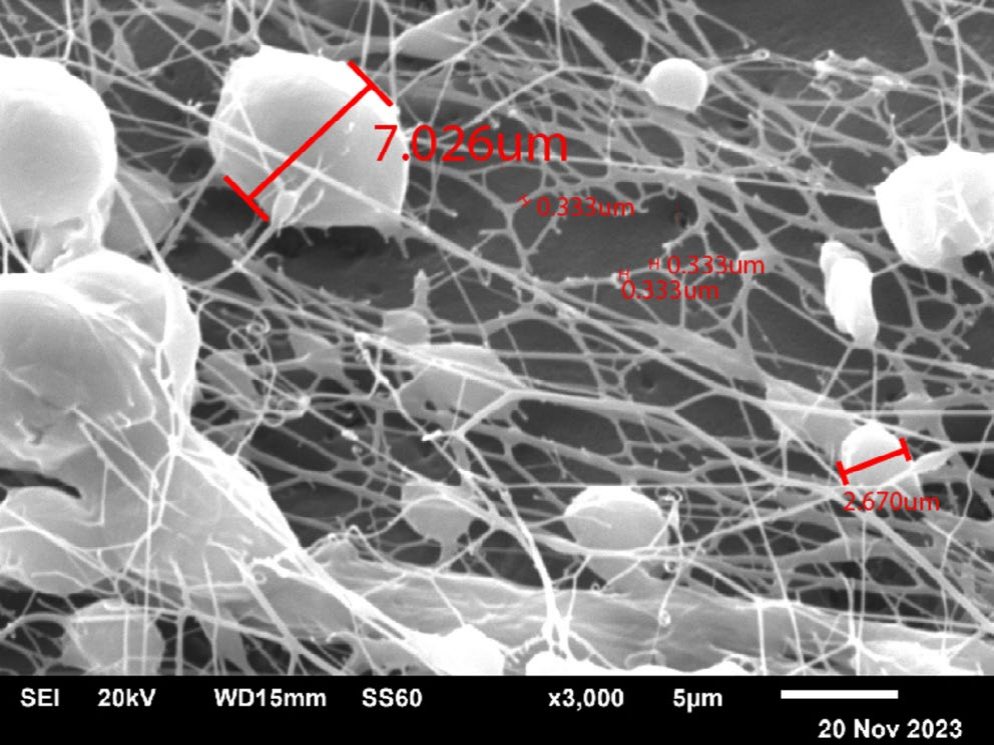
Encapsulated particles of various sizes on the surface of the threads. Scanning Electron Microscopy. Magnification ×3000.

In the second stage of this process, the medium‐sized capsule shell—sub‐microcapsules—undergoes lysis, which typically occurs within 2–3 weeks and up to the end of the third month. Moreover, hyaluronic acid creates a favorable environment for the functioning of fibroblasts in the hypodermis and dermis, regulating the formation of collagen fibers in response to thread introduction, and decreasing the probability of fibrotic changes that are confirmed by histological assessment in the below described in vivo study.

**FIGURE 3:**
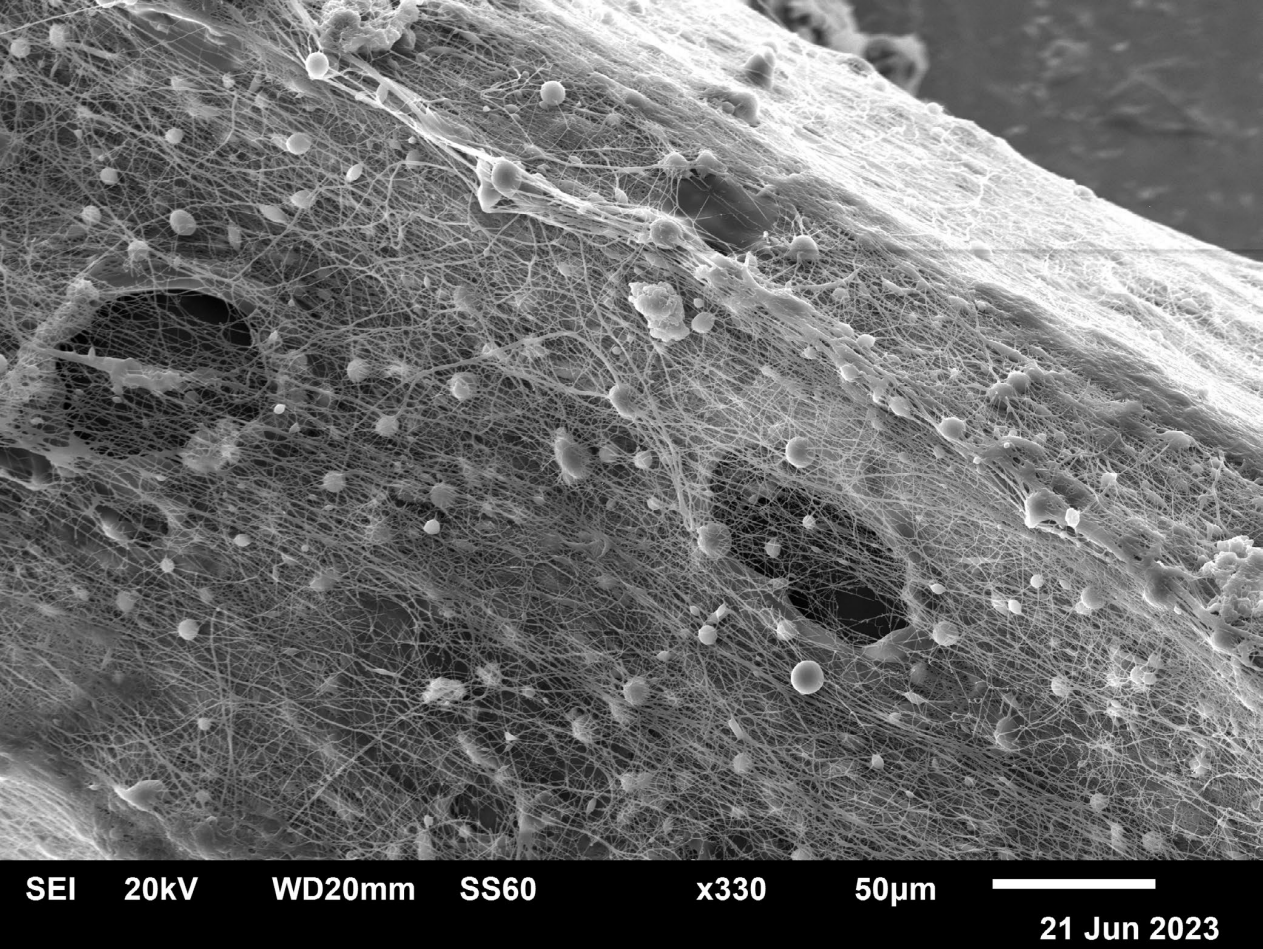
Surface structure of the thread. Scanning Electronic Microscopy. Magnification ×330.

In the final stage, the smallest particles—nanocapsules—undergo degradation. These nanocapsules have the smallest size and the maximum thickness of the capsule, ensuring that they remain on the surface of the thread for an extended period, releasing native hyaluronic acid at later stages after the thread implantation (3–6 months). The gradual release of hyaluronic acid from different size capsules allows maintaining hyaluronic acid effects for an extended time period. The gradual release of hyaluronic acid in small quantities maintains a revitalizing effect for 6 months or more and creates conditions for the optimal and physiological synthesis of collagen, prolonging the lifting effect. (Figures 1, 2, 3).

The Tensile Test Machine, Thümler GmbH TH 2730, was used to measure tensile strength, showing the threads' capability to elevate sagging tissue. Scanning Electronic Microscopy (LV‐Scanning Electron Microscope, JSM 5910 LV, Tokyo, Japan) allowed observing the unique structure of the coating, giving a prolonged rejuvenation effect.

### 2.2 Comparative Threads

The performed study utilized the packs of EV threads, consisting of 10 threads placed inside a needle 20G × 150 mm, one needle 23G × 80 mm, and one needle 18G × 40 mm in one unit, and EVHA threads, which are 10 threads coated with HA and similarly packed. Each unit was packed in a carton and sealed in foil to preserve sterility.

### 2.3 Pre‐Clinical Animal Study

The porcine model was selected due to its close resemblance to human skin in terms of structure, thickness, dermal collagen content, and healing response. Compared to rodent models, pig skin shares a similar epidermal thickness, dermal‐epidermal junction, and subcutaneous architecture with human skin [22]. This makes it particularly suitable for evaluating dermal implants, such as absorbable threads or fillers, and assessing tissue remodeling. Prior studies evaluating soft tissue fillers, including HA, PLLA, and others, have validated the porcine model's translational relevance for human dermatologic and aesthetic research [23].

Our study utilized first‐generation hybrid female pigs (Mangalitsa × Duroc) that were 5 months old and weighed between 55 and 60 kg. Animals were genetically similar siblings from the same litter. Selection criteria for participation in the experiment included a comprehensive blood test ensuring normal biochemical and hematological values, with a hemoglobin threshold of no < 110 g/L.

The nutritional regimen for the subjects was specifically designed to be low in calories. The grain components and bran of the diet underwent extrusion processing. The protein requirements were fulfilled using egg proteins and milk whey, supplemented with a comprehensive vitamin and mineral complex. This diet was formulated to cater to the unique nutritional needs of the experimental animals during the postoperative recovery phase. The exact formulation is proprietary.

The implantation technique used in this study mirrored those typically employed in human procedures [24]. It involved an initial skin puncture with a needle, followed by the introduction of the thread using a cannula from the EV product line. The thread was implanted in a single pass, avoiding any W‐shaped insertion techniques.

**FIGURE 4:**
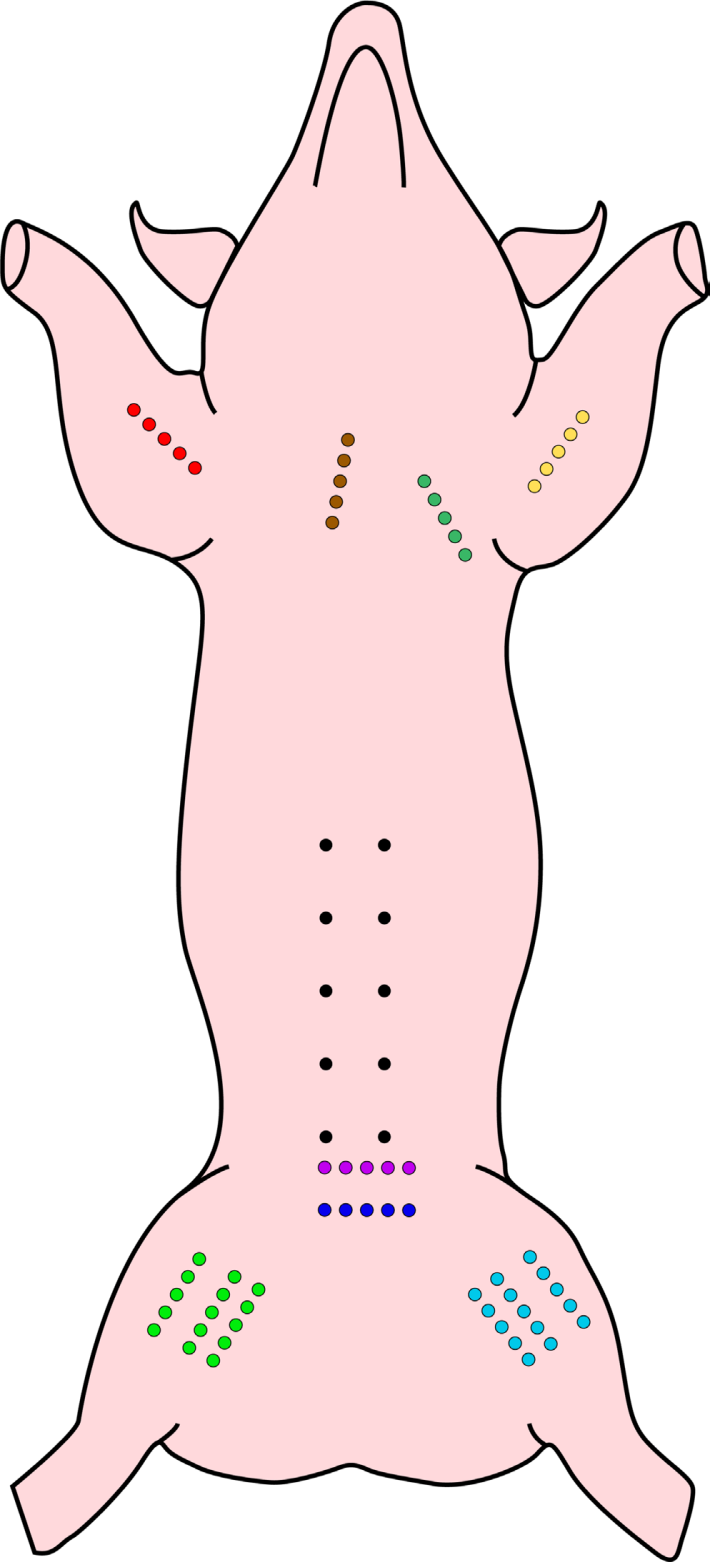
Anatomical zones for thread implantation on a pig model.

The anatomical implantation sites were specified as follows: the left surface of the thoracic cage, positioned 2 cm lateral to the media sternalis line on the left side; the right surface of the thoracic cage, located 2 cm lateral to the media sternalis line on the right; the inner surface of the front left limb, aligned with the projection of the left humerus; and the inner surface of the front right limb, corresponding to the projection of the right humerus (Figure 4).

Additionally, intact animals served as controls to provide baseline data for comparison with the experimental groups, ensuring that any observed changes could be attributed to the experimental conditions rather than natural variability in the tissue properties.

### 2.4 Histological Analyses

Histological examination was performed on a total of 15 tissue fragments. These samples were grouped based on the type of thread implanted into the subcutaneous adipose layer of the skin. The threads utilized were as follows:

1. EV
2. EVHA (with NAMICA technology)

For each type of thread, five fragments were collected at predetermined time intervals post‐implantation. The collection times were 7, 21, 30, 90, and 180 days. Thread sites were randomly distributed dorsally to avoid bias.

Histological evaluations were performed by two independent pathologists blinded to the treatment groups and time points. Tissue samples were anonymized and coded prior to microscopic assessment to ensure unbiased interpretation.

Tissue specimens were preserved using neutral 7% formalin, sequentially dehydrated in a series of alcohols with increasing concentrations, and subsequently embedded in paraffin. Sections with thicknesses ranging from 5 to 7 μm were prepared and subjected to staining protocols including hematoxylin–eosin, Weigert‐Van Gieson, and Sirius Red stains. Microscopic analysis was conducted using a Mikmed v7.1 microscope equipped with a Sony digital camera attachment, providing a resolution of 12 megapixels. A total of 3750 microphotographs were captured and evaluated. Image analysis software ImageView, Image Tool v.2.00, and GistMorph v3 were utilized to assess parameters such as inflammatory response, cellular composition, and the proportions of collagen and elastin fibers.

### 2.5 Statistical Analysis

All statistical analyses were conducted using *Statistica v.7* software. Quantitative data (e.g., collagen content, elastin fiber percentage, fibrocyte count, capsule thickness) were summarized as mean ± standard error (SE). Differences between the EV and EVHA groups at each time point were assessed using independent‐sample t‐tests. Time‐course changes within each group were evaluated using one‐way ANOVA with post hoc comparisons where applicable. A *p*‐value of < 0.05 was considered statistically significant.

## 3 Results

### 3.1 Examination of the Control Sample

#### 3.1.1 Macroscopically

No inflammation was observed in the implantation area. During the material collection, the thread was palpable and had shifted slightly from its original location. The skin and subcutaneous adipose tissue were not pathologically altered.

#### 3.1.2 Microscopically

Upon examining the histological slides stained with hematoxylin and eosin, no pathological changes in the cellular composition of the skin and subcutaneous adipose tissue were detected.

As illustrated in Table 1, in the evaluation of dermal and stromal‐vascular components, the following measurements were recorded:

**TABLE 1:** Parameters of dermis measurements and stromal‐vascular component.

Parameters	Min	Max	Average	Standard error
Dermis thickness, μm	1670.60	2211.46	1981.14	200.43
Diameter of blood vessels, μm	34.21	105.15	70.70	26.49
Relative area of vascular bed, %	2.30	9.17	5.19	2.82

Abbreviations: Max, maximum value; Min, minimum value.

The thickness of the dermis ranged from 1670.60 to 2211.46 μm, with an average thickness of 1981.14 μm. The standard error associated with the dermal thickness measurements was 200.43 μm. Blood vessel diameters within the assessed regions varied between 34.21 and 105.15 μm. The mean diameter of these vessels was found to be 70.70 μm, with a standard error of 26.49 μm. Regarding the relative area of the vascular bed, values spanned from 2.30% to 9.17%, averaging at 5.19%. The standard error for this measurement was 2.82%.

**FIGURE 5:**
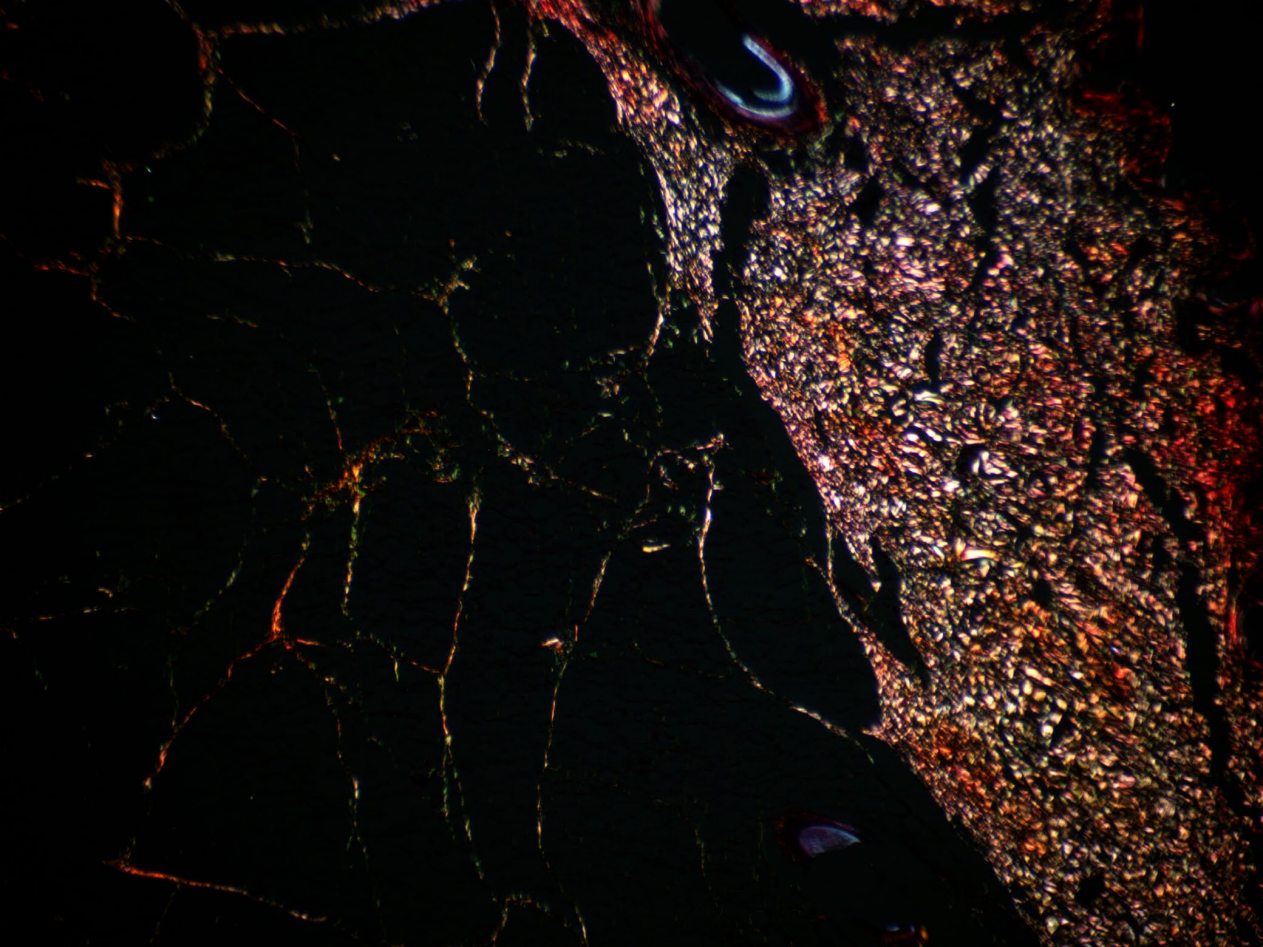
Skin and subcutaneous fat tissue. Sirius Red staining, under polarized light. ×40 magnification.

**FIGURE 6:**
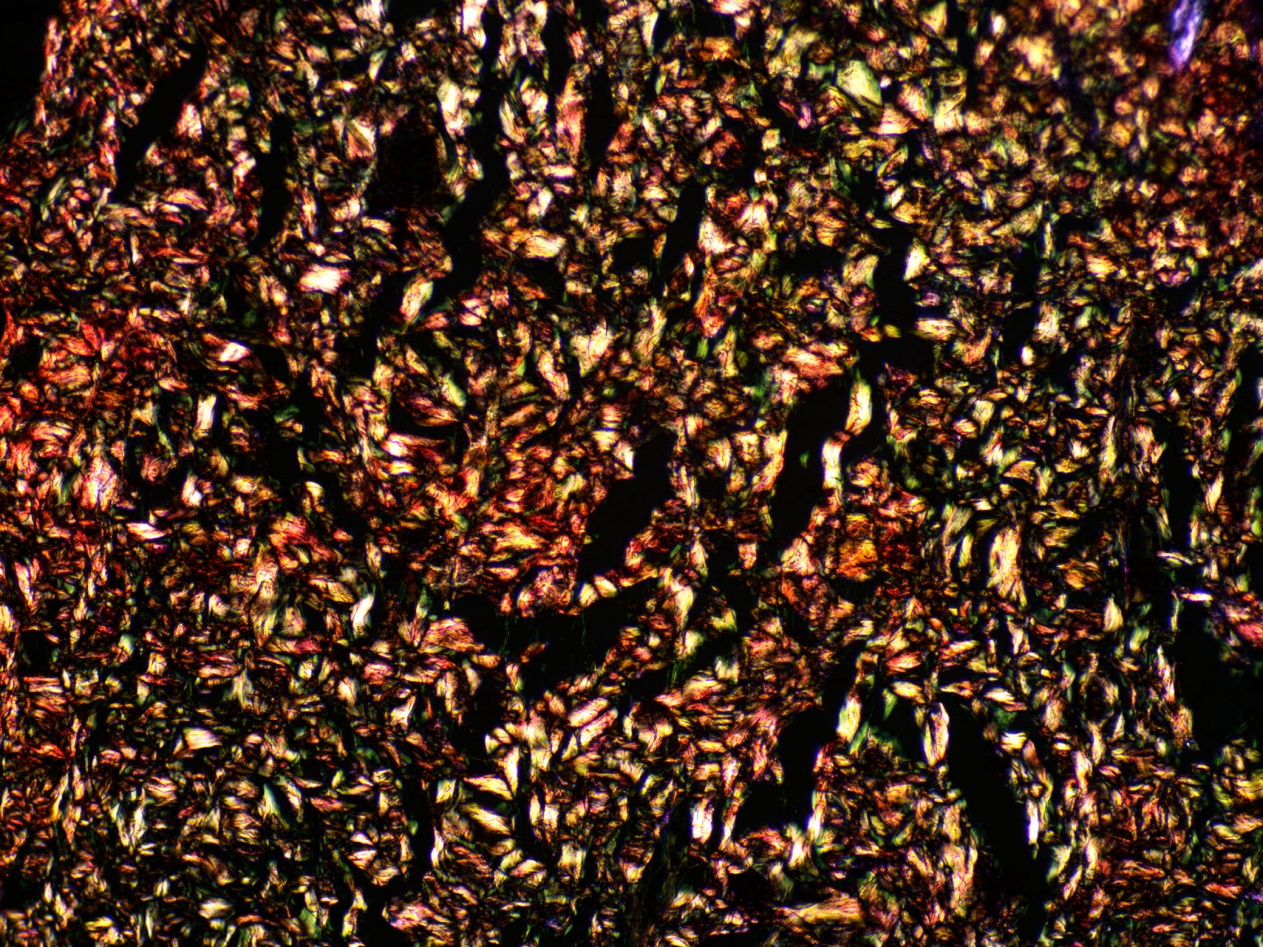
Skin. Sirius Red staining, under polarized light. ×100 magnification.

**FIGURE 7:**
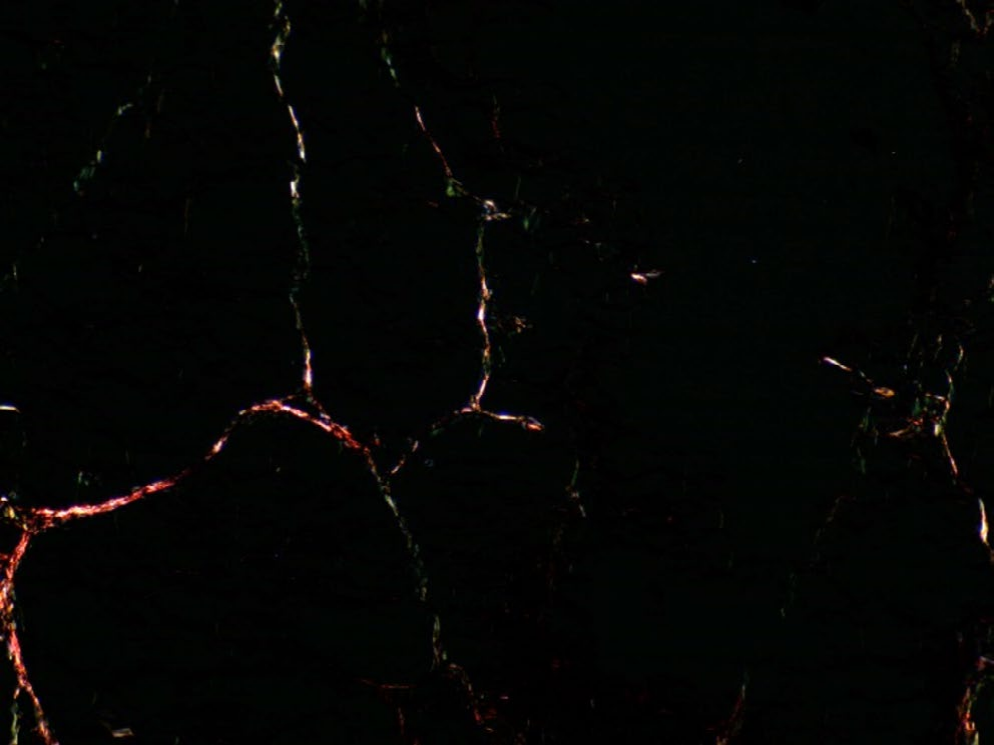
Subcutaneous fat tissue. Sirius Red staining, under polarized light. ×100 magnification.

**FIGURE 8:**
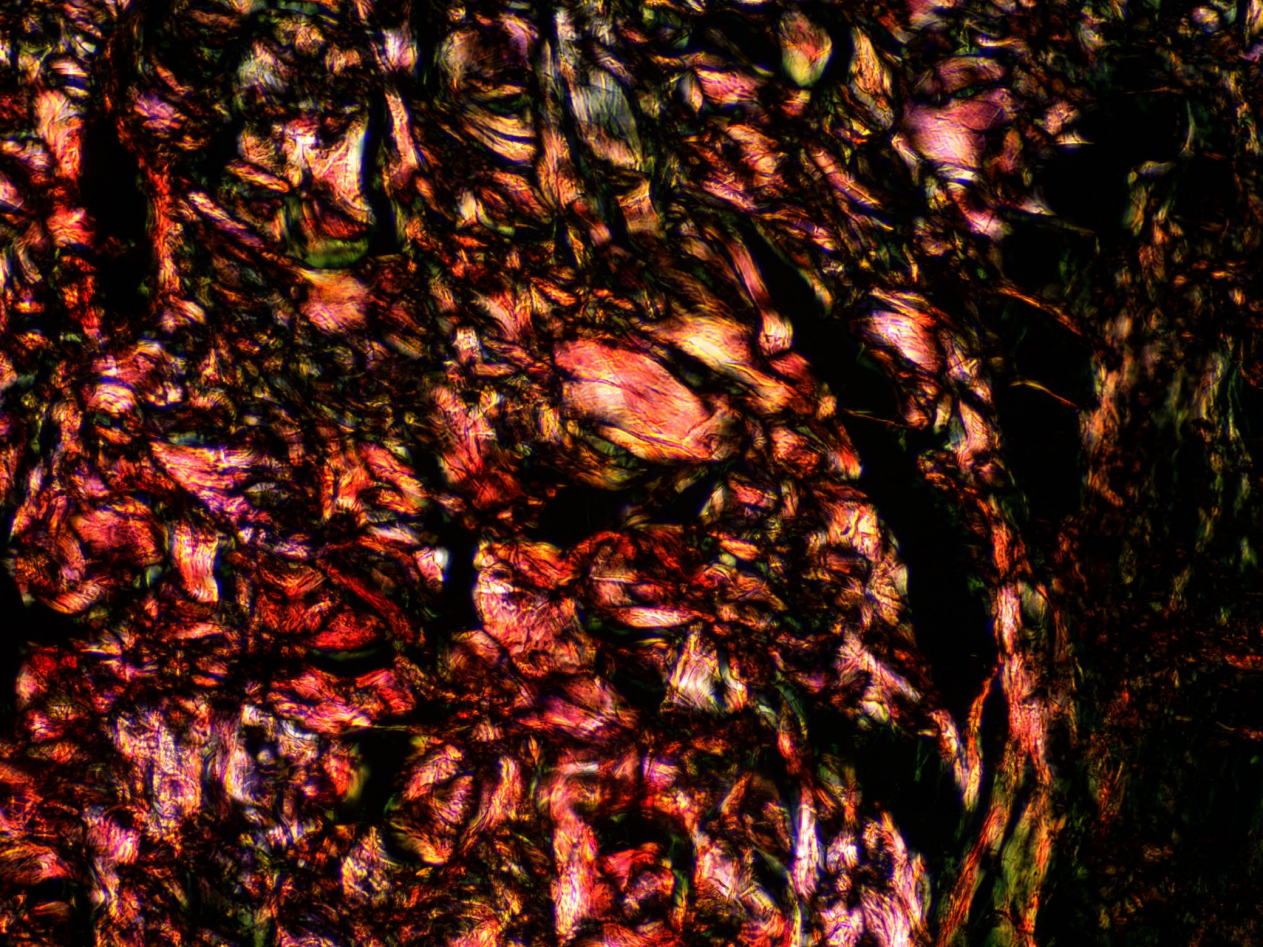
Skin. Sirius Red staining, under polarized light. ×200 magnification.

**FIGURE 9:**
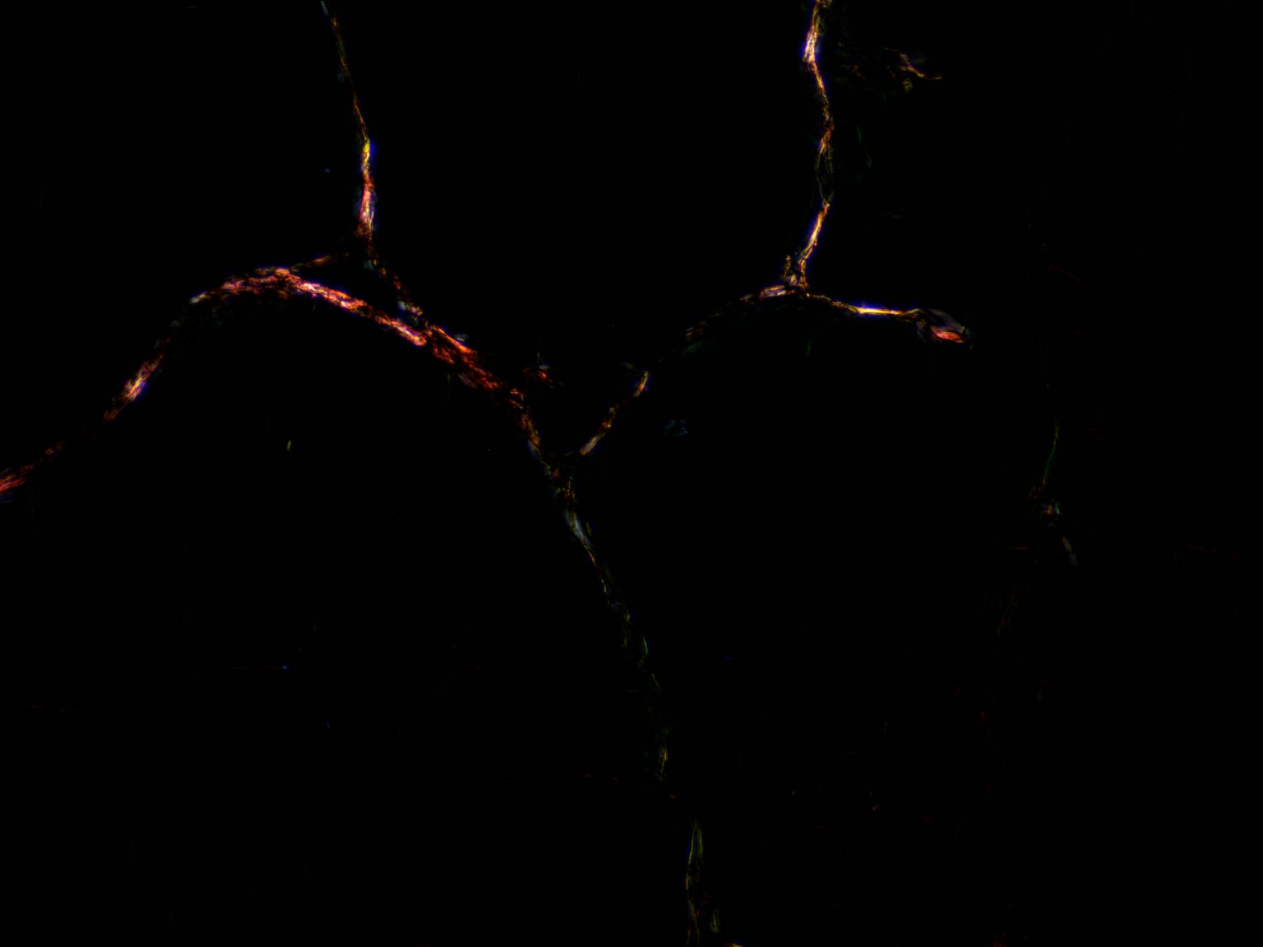
Subcutaneous fat tissue. Sirius Red staining, under polarized light. ×200 magnification.

In the slides stained using the Weigert‐Van Gieson method, the presence of elastic fibers was noted both within the dermis and in the connective tissue layers of the subcutaneous adipose tissue. When examining the histological slides stained with Sirius Red, the initial percentage ratios of type I and III collagen fibers were observed (Figures 5, 6, 7, 8, 9). Under polarized light examination, type I collagen was highlighted in the red spectrum; type III collagen was highlighted in the green spectrum.

Through spectral analysis with counting of red pixels (type I) and green pixels (type III), the following average values were obtained, shown in Table 2:

**TABLE 2:** Percentage of collagen and elastin types.

	Type of collagen	Min	Max	Average	Standard error
*Skin*					
Collagen					
Type I		55.60	85.90	71.12	13.90
TypeIII		6.10	20.70	12.18	6.20
Elastic fibers		0.90	1.50	1.14	0.23
Collagen/Elastin ratio		52.67	92.10	75.09	14.25
*Subcutaneous adipose tissue*					
Collagen					
Type I		7.30	11.70	9.20	1.69
Type III		10.80	17.50	13.70	2.65
Elastic fibers		0.80	1.70	1.10	0.35
Collagen/Elastin ratio		13.06	30.33	22.43	7.20

Abbreviations: Max, maximum value; Min, minimum value.

**FIGURE 10:**
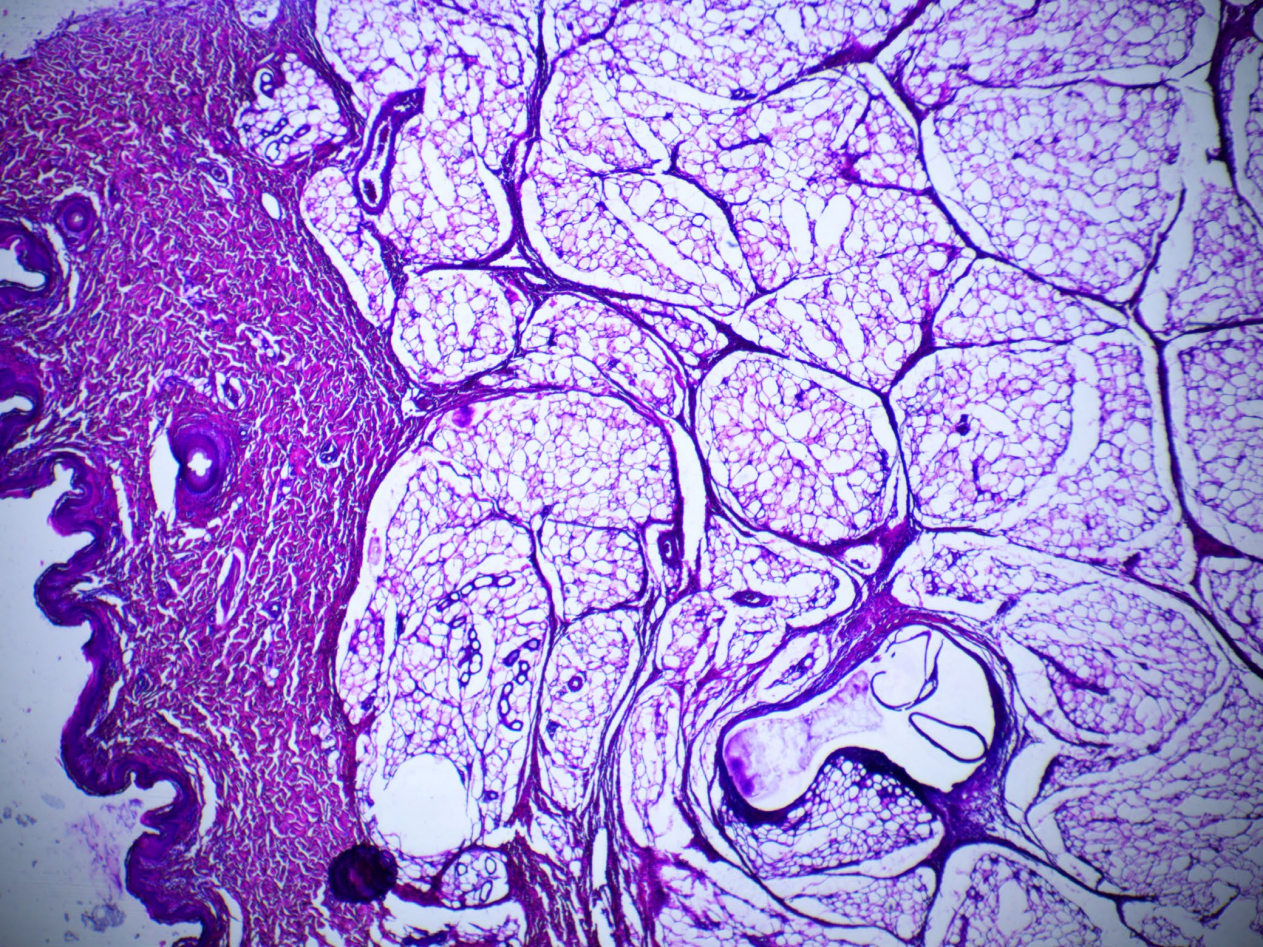
Skin and subcutaneous fat tissue. 7 days, excellence visage threads (EV). Stained with hematoxylin and eosin. ×40 magnification.

**FIGURE 11:**
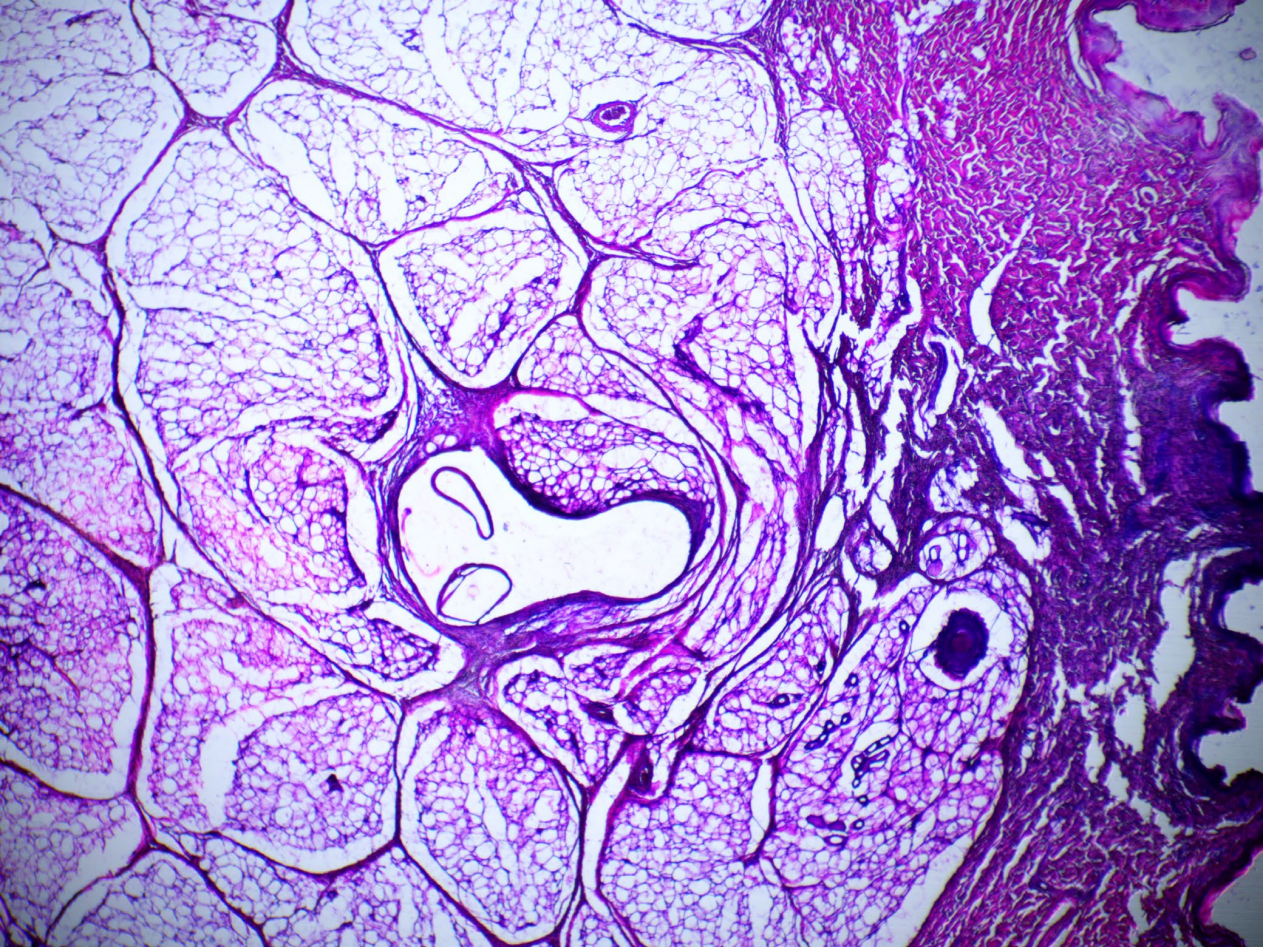
Skin and subcutaneous fat tissue. 7 days, excellence visage threads with hyaluronic acid capsules (EVHA, NAMICA technology). Stained with hematoxylin and eosin. ×40 magnification.

**FIGURE 12:**
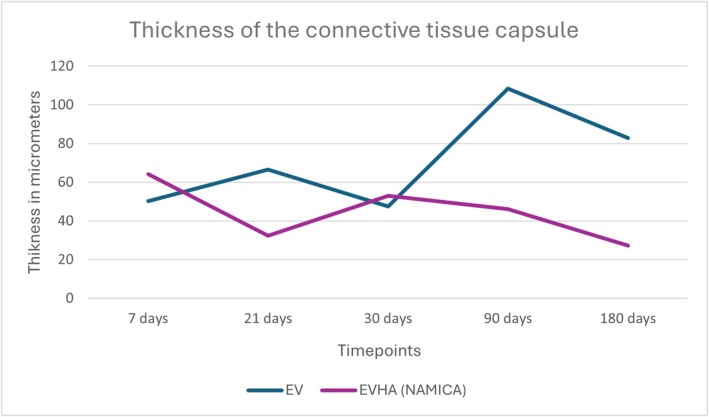
Thickness of the connective tissue capsule in micrometers. EV, excellence visage threads; EVHA, excellence visage threads with hyaluronic acid capsules.

**FIGURE 13:**
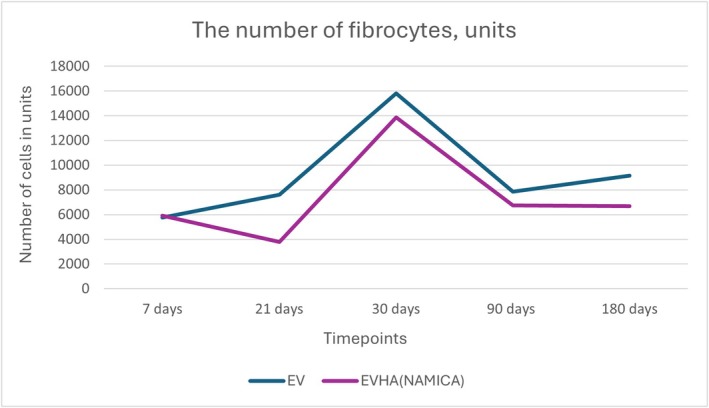
The number of fibrocytes in units. EV, excellence visage threads; EVHA, excellence visage threads with hyaluronic acid capsules (NAMICA technology).

**FIGURE 14:**
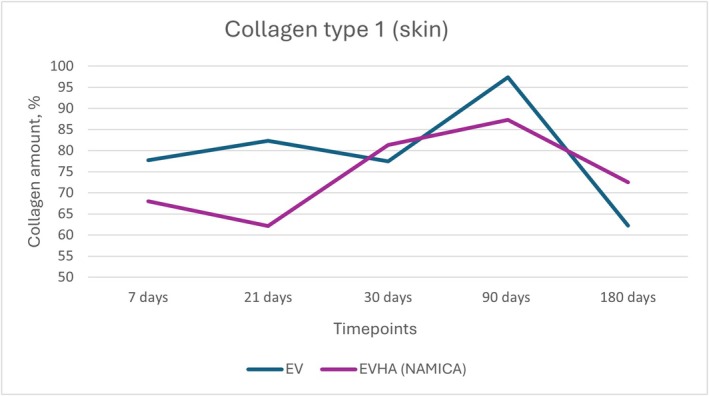
Dynamics of type I collagen synthesis in the skin following implantation of sample threads (expressed in percentages). EV, excellence visage threads; EVHA, excellence visage threads with hyaluronic acid capsules (NAMICA technology).

**FIGURE 15:**
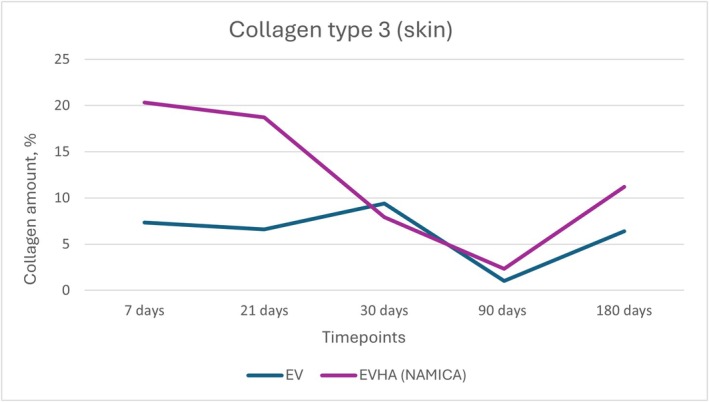
Dynamics of type III collagen synthesis in the skin following implantation of sample threads (expressed in percentages). EV, excellence visage threads; EVHA, excellence visage threads with hyaluronic acid capsules (NAMICA technology).

**FIGURE 16:**
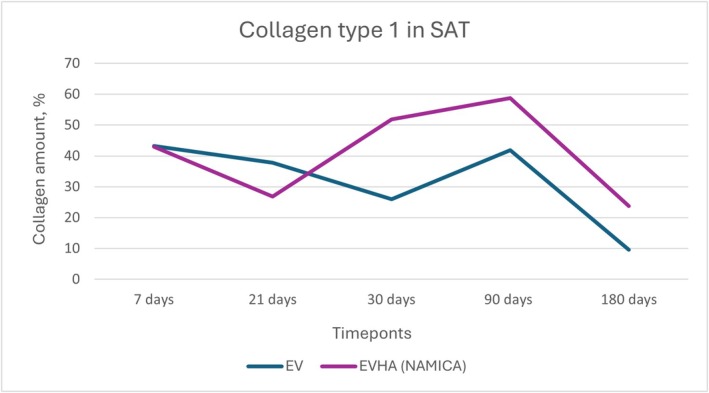
Dynamics of type I collagen synthesis in subcutaneous adipose tissue following implantation of sample threads (expressed in percentages). EV, excellence visage threads; EVHA, excellence visage threads with hyaluronic acid capsules (NAMICA technology); SAT, subcutaneous adipose tissue.

**FIGURE 17:**
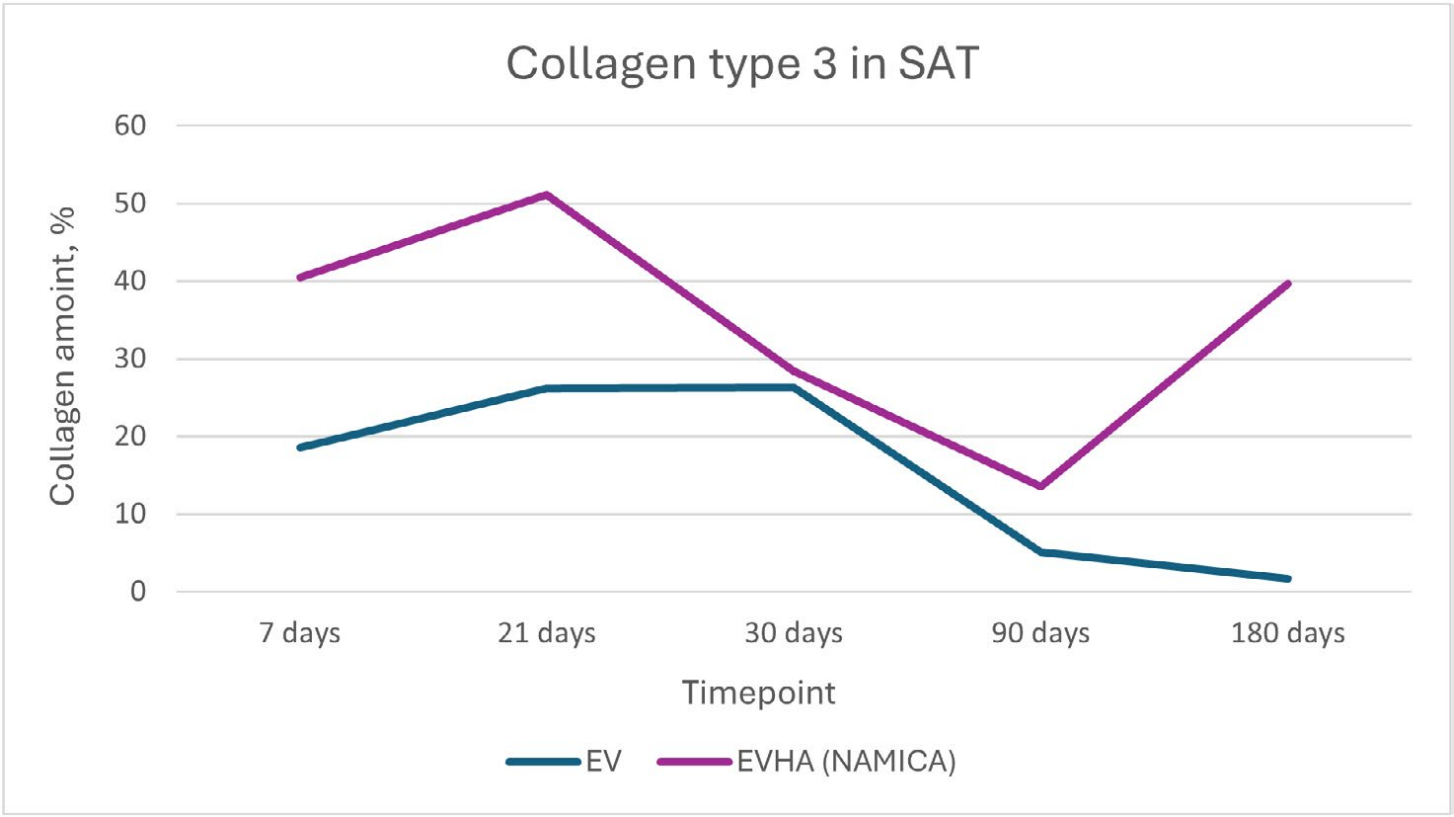
Dynamics of type III collagen synthesis in subcutaneous adipose tissue following implantation of sample threads (expressed in percentages). EV, excellence visage threads; EVHA, excellence visage threads with hyaluronic acid capsules (NAMICA technology); SAT, subcutaneous adipose tissue.

**FIGURE 18:**
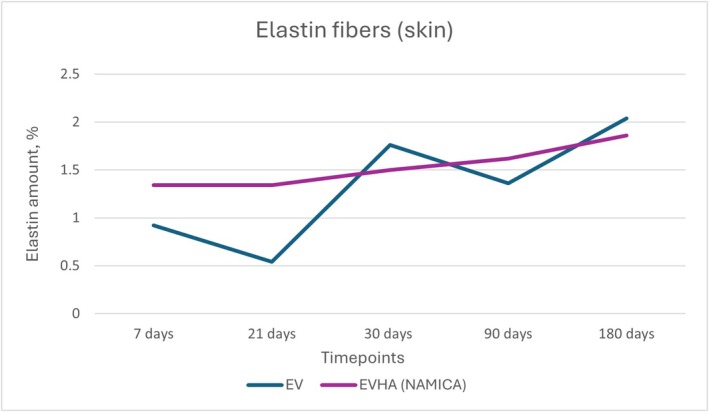
Dynamics of elastin fiber synthesis in the skin following implantation of sample threads (expressed in percentages). EV, excellence visage threads; EVHA, excellence visage threads with hyaluronic acid capsules (NAMICA technology).

**FIGURE 19:**
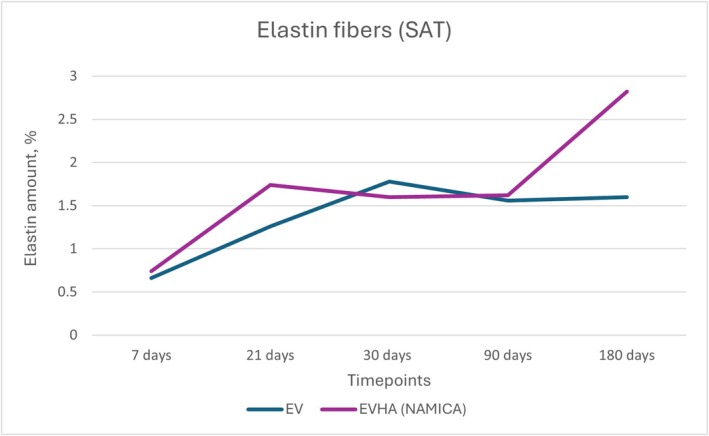
Dynamics of elastin fiber synthesis in the subcutaneous adipose tissue following implantation of sample threads (expressed in percentages). EV, excellence visage threads; EVHA, excellence visage threads with hyaluronic acid capsules (NAMICA technology); SAT, subcutaneous adipose tissue.

**FIGURE 20:**
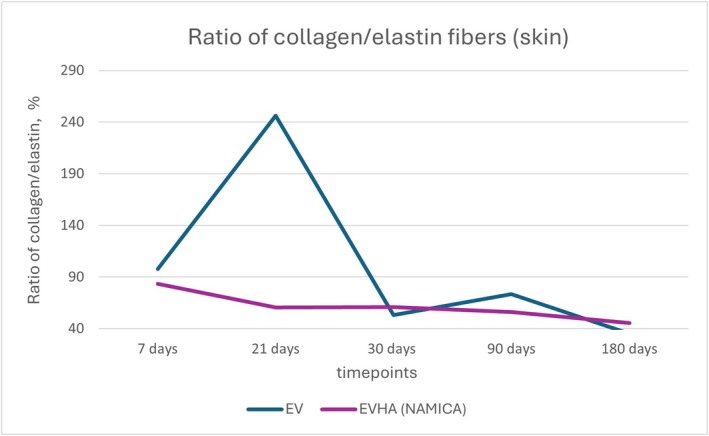
Ratio of collagen and elastin fibers in the skin following implantation of sample threads (expressed in percentages). EV, excellence visage threads; EVHA, excellence visage threads with hyaluronic acid capsules (NAMICA technology).

**FIGURE 21:**
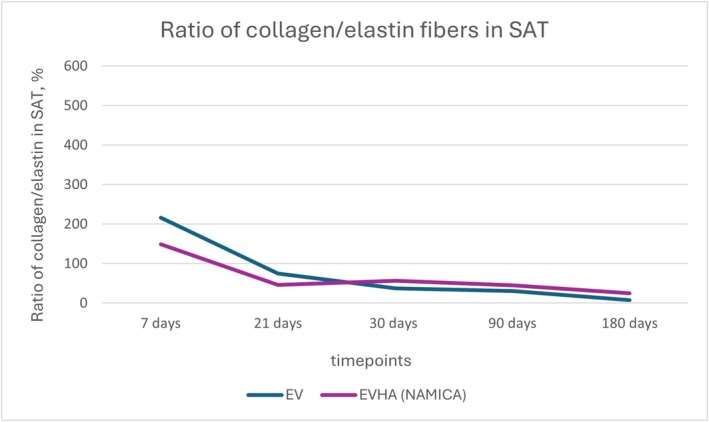
Ratio of collagen and elastin fibers in the subcutaneous adipose tissue following implantation of sample threads (expressed in percentages). EV, excellence visage threads; EVHA, excellence visage threads with hyaluronic acid capsules (NAMICA technology).

In the skin:

- Type I Collagen was observed within a range of 56.60% to 85.90%, with an average value of 71.12% and a standard error of 13.90%.
- Type III Collagen ranged from 6.10% to 20.70%, averaging 12.18% with a standard error of 6.20%.
- Elastic fibers varied from 0.90% to 1.50%, with an average of 1.14% and a standard error of 0.23%.
- The Collagen/Elastin ratio in the skin was found to be between 52.67% and 92.10%, with an average of 75.09% and a standard error of 14.25%.

In the subcutaneous adipose tissue:

- Type I Collagen percentages ranged from 7.30% to 11.70%, with an average of 9.20% and a standard error of 1.69%.
- Type III Collagen was measured between 10.80% and 17.50%, with an average of 13.70% and a standard error of 2.65%.
- Elastic Fibers were recorded between 0.80% and 1.70%, averaging 1.10% with a standard error of 0.35%.
- The Collagen to Elastin Ratio varied from 13.06% to 30.33%, averaging 22.43% with a standard error of 7.20%.

Thus, the examination of this sample revealed the control values of the parameters of the skin and subcutaneous adipose tissue.

To study the rehabilitation period and evaluate the severity of the inflammatory reaction and early effects, we conducted a histological examination of tissue samples at 7, 21, 30, 90, and 180 days after material implantation. A comparison was made with controls (intact animals) and between thread samples.

### 3.2 Assessment of the Inflammatory Reaction

In the histological evaluation of samples (EV and EVHA) at day 7 post‐implantation, no signs of acute inflammation were detected. Necrotic detritus was absent, suggesting no tissue necrosis due to mechanical damage.

Regarding cellular response, a minimal cellular reaction was observed in the EVHA sample. The cellular reaction at the implantation sites of these samples was primarily due to the presence of fibroblasts, with isolated leukocytes and macrophages (1–2 per field of view, magnification ×200) also identified. In the EV, fibroblasts were the predominant cell type, but a higher number of leukocytes and macrophages (up to 10 per field of view, magnification ×200) were observed. Neutrophils were not detected in the implantation areas. Despite the presence of these cells, the response can be considered a physiological reaction to aseptic damage and tissue regeneration. However, the reaction in the EVHA samples was milder, influenced by the presence of unstabilized hyaluronic acid, which provides additional anti‐inflammatory effects, affects the microcirculatory bed, thereby increasing the influx of interstitial fluid and enhancing tissue tropism (Figures 10 and 11).

For later implantation periods, chronic inflammation was not observed in either of the two samples. Additionally, granulomas were not detected at various post‐implantation periods, while varying degrees of collagenesis were noted. It should be noted that samples (EV, EVHA) demonstrated a progressive increase in dermal thickness over time, indicating a robust connective tissue response.

### 3.3 Thickness of the Connective Tissue Capsule

The connective tissue capsule thickness serves as a key indicator of the degree of integration between the implanted material and the body's own tissues. Within the scope of this study, the different materials showcased varying degrees of tissue compatibility and integration.

EVHA samples exhibited optimal integration, as evidenced by a wave‐like decrease in capsule thickness over time. Data regarding connective tissue capsule thickness changes over time were systematically recorded and summarized in Figure 12 with a significant reduction from 64.28 μm at 7 days to 27.23 μm by day 180. These data indicate not only high tissue compatibility but also a physiological maturation of collagen fibers within the connective tissue capsule, resulting in orderly fiber alignment.

In contrast, the EV sample demonstrated a more marked connective tissue response; capsule thickness measurements began at 50.27 μm at 7 days and showed fluctuations, with an increase to 66.53 μm at 21 days, a decrease to 47.42 μm at 30 days, a peak at 108.32 μm at 90 days, and a slight decrease to 82.93 μm at 180 days.

### 3.4 Fibrocyte Count

In evaluating the dynamics of fibrocyte populations post‐implantation, the gathered data revealed no statistically significant differences among the sample types over a 180‐day period, suggesting a common datum in the physiological response to mechanical irritation.

Fibrocyte counts over the course of 180 days are documented in Figure 13, showcasing the temporal dynamics of fibrocyte populations in response to different implant materials. Specifically, for the EV sample, initial fibrocyte counts were at 5753.42 units at 7 days, which increased to 7602.74 units by day 21, peaked at 15 821.92 units by day 30, and then decreased to 7842.47 units by day 90, with a further reduction to 9143.84 units by day 180. The EVHA sample exhibited an initial count of 5924.66 units at 7 days, a decline to 3801.37 units at 21 days, an increase to 13 869.86 units at 30 days, a subsequent decrease to 6746.58 units at 90 days, and finally a count of 6678.08 units at 180 days, demonstrating data toward stabilization albeit at higher values than initially measured.

The results at day 180 are particularly notable. The EV sample was inclined towards an increase, and the EVHA sample stabilized, maintaining higher values relative to its original count. The observation of increased fibrocytes in the EV sample could potentially indicate a propensity towards collagenosis if the data continues. The steady state of the EVHA sample supports the notion of ongoing physiological stimulation of collagen synthesis.

These findings collectively highlight a complex interplay between the implant materials and the host's tissue response, underscoring the importance of monitoring fibrocyte levels as an indicator of tissue integration and regeneration over time.

### 3.5 Collagen Type I and Type III

The measurement of Type I and Type III collagen in the skin following the implantation of various sample threads revealed an intriguing pattern of response over the course of 180 days. Collagen Type I and Type III synthesis and dynamics in the skin over 180 days is detailed in Figures 14 and 15.

In the EV sample group, initial measurements commenced at 77.76% at 7 days, with a rise to 82.34% at 21 days. There was a marginal decrease to 77.5% at 30 days, followed by a pronounced escalation to 97.36% at 90 days, and finally, a reduction to 62.2% at 180 days. The Type III collagen levels showed an initial value of 7.36% at 7 days, decreasing to 6.6% at 21 days, then increasing to 9.4% at 30 days, with a drop to 1.02% at 90 days and a rebound to 6.4% at 180 days.

EVHA sample exhibited an initial Type I collagen count of 67.98% at 7 days, which decreased to 62.16% by 21 days, increased to 81.36% at 30 days, rose further to 87.26% by 90 days, and finally leveled at 72.46% at 180 days. In contrast, the Type III collagen measurements showed a starting value of 20.34% at 7 days, a reduction to 18.72% at 21 days, an increase to 7.9% at 30 days, a decrease to 2.34% at 90 days, and an elevation to 11.22% at 180 days.

These data indicate that all samples stimulated collagen synthesis, with levels exceeding those of the control group. The data were broadly consistent across all samples, with slight variations observed.

### 3.6 Collagen Types I and III in the Subcutaneous Adipose Tissue (SAT)

In the analysis of collagen types within subcutaneous adipose tissue (SAT) post‐implantation, notable data were observed over a 180‐day study period. Collagen Type I and Type III synthesis and dynamics in the SAT over 180 days are detailed in Figures 16 and 17.

For Type III collagen within the SAT, the EV sample group commenced at 18.56% at 7 days, demonstrating an upward trend to 26.24% at 21 days, which then slightly increased to 26.32% by day 30. A significant decrease was noted at 90 days to 5.04%, ultimately leading to a further reduction to 1.66% at 180 days. In parallel, Type I collagen levels in the EV group began at 43.18%, reduced to 37.82% by day 21, and decreased further to 26% at 30 days, followed by a rise to 41.9% at 90 days and a substantial decline to 9.64% at the end of the study.

EVHA samples in SAT revealed a starting point of 40.48% for Type III collagen at 7 days, rising to 51.12% by day 21, but decreasing slightly to 28.4% by day 30. A descent to 13.5% occurred at 90 days, with a notable increase to 39.7% by day 180. For Type I collagen in the EVHA group, the levels initiated at 43% at day 7, reduced to 26.78% at 21 days, rose to 51.78% at day 30, further climbed to 58.8% at 90 days, and finally, there was a decline to 23.74% by 180 days.

These variable collagen levels across the sample groups suggest differential tissue responses induced by the implant materials. The EVHA samples displayed a consistent rise in Type III collagen, indicative of superior tissue regeneration capabilities, while the EV samples exhibited a general decline in both Type I and Type III collagen.

Collectively, the data reflect the specific effects of the implants on SAT collagen composition and provide insight into the potential of these materials for use in therapeutic and aesthetic contexts. The observed patterns indicate the capacity of these implants to modulate not only the quantity but also the quality of collagen, supporting tissue structure and elasticity, critical parameters in the application of regenerative and reconstructive treatments.

### 3.7 Elastin Fibers

In the assessment of elastin fibers in both the skin and subcutaneous adipose tissue (SAT) following the implantation of various threads, a positive influence was exerted across all three types of threads. Upon examination of the quantitative data, an increase in elastin fibers was observed. Elastin synthesis and dynamics in the skin over 180 days are detailed in Figures 18 and 19.

The EV skin samples exhibited an initial content of 0.92% on day 7, followed by a decrease to 0.54% on day 21, then a rise to 1.76% on day 30, a slight decrease to 1.36% on day 90, and a final increase to 2.04% at day 180. EVHA skin samples began with 1.34% on day 7, maintained at 1.34% on day 21, experienced a slight rise to 1.5% on day 30, increased to 1.62% on day 90, and concluded with 1.86% on day 180.

For the SAT, EV samples commenced with 0.66% at day 7, increased to 1.26% at day 21, elevated further to 1.78% at day 30, slightly decreased to 1.56% at day 90, and plateaued at 1.6% at 180 days. EVHA samples showed 0.74% at day 7, rose to 1.74% on day 21, slightly decreased to 1.6% on day 30, increased to 1.62% on day 90, and showed a significant rise to 2.82% at 180 days.

These patterns suggest that the implants positively influence elastin content, thereby enhancing tissue elasticity and structural integrity. The findings point to a tissue‐specific response that is more pronounced within the SAT, wherein the implanted materials influence the qualitative composition of elastin fibers, potentially enhancing the functional properties of the tissue. This distinction is especially relevant when considering therapeutic and aesthetic interventions aimed at tissue repair and regeneration.

In the evaluation of tissue remodeling post‐implantation, the collagen‐to‐elastin fiber ratios in the skin and subcutaneous adipose tissue (SAT) presented discernible data over a 180‐day timeline.

As detailed in Figures 20 and 21, the collagen‐to‐elastin ratio changes in skin and SAT were tracked over 180 days.

In both dermal and subcutaneous tissues, the EVHA group consistently demonstrated higher percentages of collagen type I and III compared to the EV group across all time points. Notably, at day 180, type I collagen reached 85.9% in the EVHA group versus 58.4% in the EV group, and type III collagen reached 20.7% versus 10.5%, respectively (*p* < 0.05).

Elastin content also increased progressively in EVHA‐treated sites, reaching 2.82% in SAT by day 180, compared to 1.76% in the EV group. Capsule thickness was thinner in the EVHA group across all time points (e.g., 27.23 μm vs. 64.28 μm at day 180), indicating improved tissue integration. These findings confirm that NAMICA‐coated threads not only induce stronger biostimulatory effects but also reduce fibrotic encapsulation.

The EVHA group showed notably reduced inflammatory cell infiltration at an early point, suggesting that the sustained release of native hyaluronic acid contributes to local immunomodulation. HA is known to reduce pro‐inflammatory cytokine expression, limit neutrophil infiltration, and promote M2 macrophage polarization—all of which favor regenerative healing [15, 16]. This explains the thinner, more organized capsule formation and improved fibrocyte response observed in the EVHA group compared to EV. Fibrocytes are circulating progenitor cells involved in extracellular matrix (ECM) remodeling, known to synthesize collagen and elastin during tissue repair [17]. The increase in fibrocyte numbers observed in the EVHA group aligns with enhanced regenerative remodeling. Importantly, this elevation was not accompanied by increased capsule thickness or disorganized fibrosis, indicating a balanced, controlled tissue response rather than pathological scarring.

## 4 Discussion

The present study evaluated the long‐term tissue response to P(LA/CL)‐HA threads manufactured with NAMICA technology in a porcine model over a 180‐day period. Our results demonstrate that these threads induce sustained collagen synthesis, elastin production, and a gradual, wave‐like decrease in fibrotic capsule thickness—indicating a favorable integration profile and reduced risk of chronic fibrosis. The effects and properties of PLLA and polycaprolactone (PCL), administered as lifting threads or solutions, are being studied with a focus on their collagen‐stimulating abilities [19, 20, 25, 26]. The study by Bernardo et al. demonstrated no significant increase in collagen type I content and some increase in type III collagen in rats 1 month after injection of PLLA solution [25]. Our findings differ, as we observed a progressive increase in collagen type I beginning after the first month and continuing through day 180 following the insertion of solid P(LA/CL) threads. While this discrepancy may be partly attributed to thread morphology versus injectable formulation, the choice of animal model likely played a key role. Unlike rodents, pigs offer a closer approximation to human skin in terms of thickness, dermal structure, and healing response, and the longer observation period in our study strengthens the evidence for a time‐dependent increase in collagen type I. Additionally, Bernardo's report of disorganized collagen fibers surrounding PLLA injections contrasts with our findings of more structured and uniformly aligned extracellular matrix architecture. This improvement may be attributed to the controlled release of hyaluronic acid (HA) from NAMICA‐coated threads, which modulates the inflammatory response and promotes regenerative matrix remodeling.

A pivotal study by Burko et al. investigated nano‐ and microencapsulated HA in P(LA/CL) threads using a comparable porcine model. Their findings highlighted that nano‐sized HA particles enabled a sustained, bioactive release profile, which significantly enhanced hydration, neocollagenesis, and vascularization over 180 days. The absence of adverse fibrotic responses, coupled with improved dermal architecture, supports our observation of reduced capsule thickness and steady increases in collagen and elastin across all time points [26].

Furthermore, two studies published by Burko et al. specifically compared HA‐coated and non‐coated P(LA/CL) threads in a porcine model over the same 180‐day timeframe. Data confirmed that HA‐coated threads induced significantly greater collagen deposition, elastin fiber production, and tissue vascularity than uncoated threads. Notably, their HA threads yielded a more structured and integrated collagen matrix with minimal chronic inflammation—paralleling our findings of a milder inflammatory response, more physiological fibrocyte behavior, and superior collagen I/III dynamics in EVHA samples. This convergence across studies reinforces the synergistic effect of combining P(LA/CL) copolymer with bioavailable HA via controlled release technology, such as NAMICA, in aesthetic medicine applications [27, 28].

## 5 Conclusion

The preclinical evaluation of P(LA/CL)‐HA threads enhanced with NAMICA technology has revealed substantial benefits in the field of skin rejuvenation. This cutting‐edge approach not only provides mechanical lifting but also ensures a prolonged release of hyaluronic acid, significantly boosting skin hydration and collagen synthesis. These threads effectively minimize inflammatory responses, a common concern with rejuvenation therapies, while promoting superior tissue integration and increased collagen density. Histological studies confirm these benefits, suggesting that the threads can deliver both immediate improvements in skin aesthetics and enduring benefits by enhancing the underlying skin structure over a 6‐month redout.

These promising results highlight the threads' potential to redefine therapeutic standards in non‐surgical aesthetic treatments, emphasizing their ability to meet the growing demand for less invasive yet effective cosmetic solutions. The success of these threads in preclinical trials makes a compelling case for subsequent clinical studies. These studies are essential to comprehensively assess the efficacy and safety of the P(LA/CL)‐HA threads with NAMICA technology, potentially establishing a new benchmark in aesthetic medicine. Such research could ultimately lead to widespread adoption and optimization of thread lift techniques, offering patients innovative and reliable options for skin rejuvenation.

## Author Contributions

D.N. designed the study and carried out experiments; G.S. supervised the study and revised the manuscript; A.K. analyzed the data and revised the manuscript. All authors had access to relevant data and participated in the review and approval of this publication.

## Ethics Statement

All animal procedures including housing and experimental procedures were performed in strict accordance with corresponding ethical principles and norms. The study was approved by the Ethics Committee of the Preclinical Research Center, Penza, Russian Federation (No. 5‐2023, 23 August 2023).

## Consent

The authors have nothing to report.

## Conflicts of Interest

A.K. is a scientific consultant at Aptos LLC. G.S. has an ownership interest in medical products and techniques of Aptos LLC. D.N. is employed at Aptos LLC. The employment and ownership relationships have neither any interference with the activities and the results of this study nor the ability of the authors to process the reported material fairly, rationally, and without any kind of bias.

## Data Availability

The data supporting the findings of this study are available from the corresponding author upon reasonable request.

## References

[1] J. K. Song , J. Chang , K. W. Cho , and C. Y. Choi , “Favorable Crisscrossing Pattern With Polydioxanone: Barbed Thread Lifting in Constructing Fibrous Architecture,” Aesthetic Surgery Journal 41, no. 7 (2021): 875–886. 10.1093/asj/sjab153 33784374

[2] N. S. Sadick , S. Manhas‐Bhutani , and N. Krueger , “A Novel Approach to Structural Facial Volume Replacement,” Aesthetic Plastic Surgery 37, no. 2 (2013): 266–276. 23358580 10.1007/s00266-012-0052-6

[3] M. Litwiniuk , A. Krejner , and T. Grzela , “Hyaluronic Acid in Inflammation and Tissue Regeneration,” Wounds: a Compendium of Clinical Research and Practice 28, no. 3 (2016): 78–88. 26978861

[4] J. Liang , D. Jiang , J. Griffith , et al., “CD44 Is a Negative Regulator of Acute Pulmonary Inflammation and Lipopolysaccharide‐TLR Signaling in Mouse Macrophages,” Journal of Immunology 178, no. 4 (2007): 2469–2475. 10.4049/jimmunol.178.4.2469 17277154

[5] K. Bohnert , A. Dorizas , P. Lorenc , and N. S. Sadick , “Randomized, Controlled, Multicentered, Double‐Blind Investigation of Injectable Poly‐l‐Lactic Acid for Improving Skin Quality,” Dermatologic Surgery 45, no. 5 (2019): 718–724. 30741790 10.1097/DSS.0000000000001772

[6] M. T. Aznabaev , A. R. Imaeva , S. A. Bashkatov , and A. F. Gabdrakhmanova , “Anti‐Inflammatory Activity of Hyaluronic Acid” [in Russian], Experimental and Clinical Pharmacology 66, no. 5 (2003): 28–29. 14650211

[7] N. M. Salwowska , K. A. Bebenek , D. A. Żądło , and D. L. Wcisło‐Dziadecka , “Physiochemical Properties and Application of Hyaluronic Acid: A Systematic Review,” Journal of Cosmetic Dermatology 15, no. 4 (2016): 520–526. 27324942 10.1111/jocd.12237

[8] A. da Costa , D. G. Z. Biccigo , E. T. de Souza Weimann , et al., “Durability of Three Different Types of Hyaluronic Acid Fillers in Skin: Are There Differences Among Biphasic, Monophasic Monodensified, and Monophasic Polydensified Products?,” Aesthetic Surgery Journal 37, no. 5 (2017): 573–581. 27923810 10.1093/asj/sjw161

[9] A. Cazzaniga , A. C. Ballin , and F. S. Brandt , “Long‐Term Efficacy, Safety and Durability of Juvéderm XC,” Clinical, Cosmetic and Investigational Dermatology 6 (2013): 183–189. 23946665 10.2147/CCID.S33568 PMC3739705

[10] M. N. Ave and M. C. de Almeida Issa , “Hyaluronic Acid Dermal Filler: Physical Properties and Its Indications,” in Botulinum Toxins, Fillers and Related Substances. Clinical Approaches and Procedures in Cosmetic Dermatology, ed. M. Issa and B. Tamura (Springer, 2018), 1–11.

[11] M. Li , J. Sun , W. Zhang , Y. Zhao , S. Zhang , and S. Zhang , “Drug Delivery Systems Based on CD44‐Targeted Glycosaminoglycans for Cancer Therapy,” Carbohydrate Polymers 251 (2021): 117103. 33142641 10.1016/j.carbpol.2020.117103

[12] A. Fallacara , S. Manfredini , E. Durini , and S. Vertuani , “Hyaluronic Acid Fillers in Soft Tissue Regeneration,” Facial Plastic Surgery 33 (2017): 87–96. 28226376 10.1055/s-0036-1597685

[13] S. G. Moillard , J. B. Betemps , B. Hadjab , D. Topchian , P. Micheels , and D. Salomon , “Key Rheological Properties of Hyaluronic Acid Fillers: From Tissue Integration to Product Degradation,” Plastic and Aesthetic Research 5 (2018): 17–24.

[14] S. L. Basta , “Cosmetic Fillers Perspective on the Industry,” Facial Plastic Surgery Clinics of North America 23 (2015): 417–421. 26505538 10.1016/j.fsc.2015.07.001

[15] R. Stern , A. A. Asari , and K. N. Sugahara , “Hyaluronan Fragments: An Information‐Rich System,” European Journal of Cell Biology 85, no. 8 (2006): 699–715, 10.1016/j.ejcb.2006.05.009. 16822580

[16] G. M. Campo , A. Avenoso , G. Nastasi , et al., “Hyaluronan Reduces Inflammation in Experimental Arthritis by Modulating TLR‐2 and TLR‐4 Cartilage Expression,” Biochimica et Biophysica Acta (BBA)‐Molecular Basis of Disease 1812, no. 9 (2011): 1170–1181, 10.1016/j.bbadis.2011.06.006. 21723389

[17] J. W. Reinhardt and C. K. Breuer , “Fibrocytes: A Critical Review and Practical Guide,” Frontiers in Immunology 12 (2021): 784401, 10.3389/fimmu.2021.784401. 34975874 PMC8718395

[18] F. Niforos , R. Acquilla , P. Ogilvie , et al., “A Prospective, Open‐Label Study of Hyaluronic Acid‐Based Filler With Lidocaine (VYC‐15L) Treatment for the Correction of Infraorbital Skin Depressions,” Dermatologic Surgery 43, no. 10 (2017): 1271–1280. 28858926 10.1097/DSS.0000000000001127

[19] Y. I. Ha , J. H. Kim , and E. S. Park , “Histological and Molecular Biological Analysis on the Reaction of Absorbable Thread; Polydioxanone and Polycaprolactone in Rat Model,” Journal of Cosmetic Dermatology 21, no. 7 (2022): 2774–2782. 34847267 10.1111/jocd.14587

[20] S. W. Cho , B. H. Shin , C. Y. Heo , and J. H. Shim , “Efficacy Study of the New Polycaprolactone Thread Compared With Other Commercialized Threads in a Murine Model,” Journal of Cosmetic Dermatology 20, no. 9 (2021): 2743–2749. 33421303 10.1111/jocd.13883 PMC8451902

[21] G. W. Hong , S. B. Kim , S. Y. Park , J. Wan , and K. H. Yi , “Basic Concepts in Facial and Neck Thread Lifting Procedures,” Skin Research and Technology 30, no. 4 (2024): e13673. 38584590 10.1111/srt.13673 PMC10999942

[22] G. W. Hong , S. B. Kim , S. Y. Park , J. Wan , and K. H. Yi , “Thread Lifting Materials: A Review of Its Difference in Terms of Technical and Mechanical Perspective,” Clinical, Cosmetic and Investigational Dermatology 17 (2024): 999–1006. 38737945 10.2147/CCID.S457352 PMC11086642

[23] K. H. Yi , “What Are Filling (Volumizing) Threads?,” Skin Research and Technology 30, no. 3 (2024): e13658. 38514895 10.1111/srt.13658 PMC10957715

[24] M. Sulamanidze , G. Sulamanidze , and C. Sulamanidze , “Elimination of Aesthetic Deformations of the Midface Area Our Experience,” Aesthetic Plastic Surgery 42, no. 3 (2018): 774–790. 29532106 10.1007/s00266-018-1112-3

[25] R. T. R. Bernardo , R. C. G. Oliveira , K. M. S. Freitas , J. R. Albergaria‐Barbosa , and C. M. Rizzatti‐Barbosa , “Effect of Poly‐l‐Lactic Acid and Polydioxanone Biostimulators on Type I and III Collagen Biosynthesis,” Skin Research and Technology 30, no. 4 (2024): e13681. 38584576 10.1111/srt.13681 PMC10999943

[26] P. Burko , G. Sulamanidze , and D. Nikishin , “NAMICA Encapsulation Technology in an Animal Model: MICROscale vs. NANOscale Hyaluronic Acid Particles in Skin Remodeling (Part 2),” Cosmetics 12, no. 2 (2025): 55, 10.3390/cosmetics12020055.

[27] P. Burko , G. Sulamanidze , and D. Nikishin , “Efficacy of Lifting Threads Composed of Poly(l‐Lactide‐Co‐ε‐Caprolactone) Copolymers Coated With Hyaluronic Acid: A Long‐Term Study on Biorevitalizing Properties in Skin Remodeling,” Journal of Cosmetic Dermatology 24, no. 3 (2025), 10.1111/jocd.70077. PMC11898156 40071656

[28] P. Burko , G. Sulamanidze , and D. Nikishin , “Long‐Term Efficacy of Poly(l‐Lactide‐Co‐ε‐Caprolactone) Copolymer Lifting Threads With Encapsulated MICROscale Hyaluronic Acid Particles Using NAMICA Technology: Investigating Biorevitalizing Effects in Skin Remodeling (Part 1),” Cosmetics 12, no. 1 (2025): 20, 10.3390/cosmetics12010020.

